# A multicenter, international, randomized, single-blind, placebo-controlled study of the efficacy and safety of inosine-nicotinamide-riboflavin-succinic acid in the acute period of traumatic brain injury in adults

**DOI:** 10.3389/fneur.2025.1683976

**Published:** 2026-01-02

**Authors:** Tatiana Kharitonova, Tatiana Bragina, Ekaterina Ivanova, Alexander Savello, Taras Skoromets, Sergey Petrikov

**Affiliations:** 1 National Society of Neurosonology and Cerebral Circulation, Saint Petersburg, Russia; 2 Department of Anaesthesiology and Critical Care, Ivanovo Regional Clinical Hospital, Ivanovo, Russia; 3 Department of Biostatistics, IPHARMA LLC (ChemRar Group), Moscow, Russia; 4 Department of Neurosurgery, S.M. Kirov Military Medical Academy of the Ministry of Defense of the Russian Federation, Saint Petersburg, Russia; 5 Department of Neurosurgery, First St. Petersburg I.P. Pavlov State Medical University, Saint Petersburg, Russia; 6 State Budgetary Healthcare Institution “N.V. Sklifosovsky Research Institute for Emergency Medicine of the Moscow City Health Department”, Moscow, Russia

**Keywords:** traumatic brain injury, brain contusion, neuroprotection, antioxidant, succinic acid, inosine, nicotinamide, riboflavin

## Abstract

**Background:**

The combination of inosine, nicotinamide, riboflavin, and succinic acid (INRSA) has previously demonstrated neuroprotective effect. We aimed to study the efficacy and safety of treatment with INRSA in traumatic brain injury (TBI).

**Materials and methods:**

In our multicenter, randomized, placebo-controlled, single-blind clinical trial, we studied TBI patients aged 18–60, diagnosed with cerebral contusion without compression, admission Glasgow Coma Scale (GCS) score of 13–14, and posttraumatic amnesia. The INRSA/placebo was administered intravenously twice daily for 10 days. The primary endpoints were the Galveston Orientation and Amnesia Test (performed on days 2–14), and Glasgow Outcome Scale–Extended (GOS-E; assessed on day 90 via telephone interview).

**Results:**

Between November 2020 and May 2024, 166 patients were enrolled, median age 43 (IQR 33–51), 113 (68.5%) males, all with admission GCS 13–14, 123 (74.5%) had closed-type TBI. In the interim analysis, complete recovery (GOS-E category 8) was achieved in 24 (30%) patients in the placebo group and 55 (73%) in the INRSA group. The difference in proportions was 44% (95% CI 0.26; 0.62); experimental treatment increased the odds of complete recovery by 6.53 (95% CI 3.30–13.41) compared to placebo. Based on the pre-specified criteria, the independent data monitoring committee recommended termination of the study due to early demonstration of efficacy by one of the two primary endpoints.

**Conclusion:**

Treatment with INRSA was associated with improvement in mild TBI outcome, which represents a new potential option for the pharmacological treatment of non-fatal head injury. Further studies might be of research interest and clinical demand.

**Clinical trial registration:**

ClinicalTrials.gov, identifier: NCT04631484; unique protocol ID: CTF-III-CCT-2019.

## 1 Introduction

Non-fatal traumatic brain injury (TBI) constitutes the majority of head trauma and may cause long-term decline of work capacity and functioning in daily living (1–3). Although the consequences of mild to moderate TBI involve primarily cognitive and behavioral aspects and may not be considered severe, they affect a huge population due to high TBI prevalence, especially in developing countries (4, 5).

Proven strategies to prevent these sequelae are currently lacking (1, 6), since well-designed studies of pharmacological treatments in mild TBI seem underrepresented. While rehabilitation offers a variety of options (7), neuroprotection in TBI is yet to be developed and proven in successful clinical trials (8).

Various mechanisms may play a role in the pathogenesis of secondary injury in non-fatal TBI, including transient impairment of local cerebrovascular reactivity, with an initial reduction in cerebral blood flow for hours to days (9). Addressing the cerebral ischemia, the drug combination of inosine, nicotinamide, riboflavin, and succinic acid (INRSA; manufactured as ≪Cytoflavin≫ by POLYSAN Ltd., Russia), has been previously used in acute stroke in routine clinical practice in several countries as an antihypoxant and antioxidant in order to improve outcome (10). The combination has demonstrated evidence of antioxidant effect in hemorrhagic stroke (11) and reduction of symptoms of diabetic neuropathy (12) with an excellent safety profile. In experimental TBI, INRSA was associated with the reduction of oxidative stress and lipid peroxidation and improvement of pro- and antioxidant balance in rats (13, 14). The authors conclude that INRSA may potentially contribute to the reduction of secondary damage during the post-traumatic period.

We aimed to study the efficacy and safety of treatment with INRSA added to the standard therapy in patients with mild-to-moderate TBI, defined as a Glasgow coma scale (GCS) score of 9–14 without CT evidence of mass effect at the time of inclusion.

## 2 Materials and methods

### 2.1 Study design

This study was an international, multicenter, randomized, placebo-controlled, single-blind, independent blinded outcome assessment, parallel-group clinical trial with an adaptive design. The study was conducted at 16 hospitals in Russia and one hospital in Kazakhstan (clinicaltrials.gov identifier: NCT04631484) in accordance with the Guidelines for Good Clinical Practice of the International Council for Harmonization (ICH GCP) and the Eurasian Economic Union, the principles specified in the Declaration of Helsinki, and the current legislation of the Russian Federation and Kazakhstan.

The study protocol was initially approved by the Russian Ministry of Health (no. 148) on April 02, 2020 (protocol version 1.0, amended on February 25, 2020). During the study, the primary endpoint was modified (protocol version 6.0 dated August 24, 2023, and version 6.1 for the Republic of Kazakhstan dated November 13, 2023), and the data analysis plan was adjusted accordingly. All amendments were approved by the Russian Ministry of Health, the Central Clinical Hospital, and the National Center for Expertise in Medicine of the Republic of Kazakhstan.

Prior to the start of the trial, approval from the local ethics committee was obtained from all study sites. The participants provided written informed consent prior to recruitment. The treating neurosurgeon, by the decision of a council of three physicians, could proxy consent for patients who were unable to sign the form due to impairment of consciousness, provided that the patient's consent had to be obtained immediately after reaching a conscious state.

The main inclusion criteria were age 18–60; cerebral contusion of moderate severity without compression; GCS on inclusion 9–14; initiation of treatment within 24 h after the injury; presence of post-traumatic amnesia, confusion and/or disorientation; absence of indications for a surgical intervention under general anesthesia.

The main exclusion criteria were surgical intervention under general anesthesia; polytrauma, except concomitant injuries of the skeleton, soft tissues, and internal organs, which by themselves did not require in-hospital treatment; penetrating open TBI; presence of intracranial hematoma or hemorrhagic focal contusions; severe hypoxia/hypotension/hypothermia; respiratory failure; unconsciousness caused by medications or substance abuse; aphasia/language barrier; status epilepticus; or post-ictus.

A complete list of the eligibility criteria is provided in the online Supplementary material.

### 2.2 Randomization and blinding

Patients were randomized to the INRSA/placebo group at a ratio of 1:1, controlled by the Interactive Web Response System (IWRS). Block randomization (block size = 4) was stratified by GCS score on screening (9–12 vs. 13–14); each stratum was required to comprise at least 35% of the sample to ensure balanced representation and avoid disproportionately small or large strata. One interim analysis was planned, at which point the smallest stratum had to account for at least 35% and the largest no more than 65% of the enrolled patients. The randomization list was generated by an independent statistician.

The study medication is a transparent liquid colored yellow by riboflavin. Owing to the impossibility of preparing a placebo solution similar in color, a single-blind study design was chosen. Masking methods included a dark bag on the vial, an opaque infusion system; and the preparation of the solution was hidden from the patient. The Galveston Orientation and Amnesia Test (GOAT) assessments were performed by an independent assessor who was blinded to the assigned treatment.

A prespecified interim analysis was planned at 50% enrollment. To conduct the interim data analysis, an independent data monitoring committee (DMC) of four committee members was formed, including two neurosurgeons, an independent medical adviser, and an independent biostatistician. Standard operating procedures (SOPs) and regulations of the DMC work were developed to ensure blinding of the sponsor and the study team until the study completion. Throughout the study and until the final analysis, all DMC documentation was stored with strictly limited access. While working with data, only unblinded employees of the clinical team interacted with the DMC.

### 2.3 Outcomes

Initially, the primary outcome measure was the proportion of patients with regression of post-traumatic amnesia (PTA) by day 7 of treatment, defined as having a GOAT (15) of >75 points on three consecutive days. However, as demonstrated by actual patient recruitment, not enough patients with a GCS score of 9–12, corresponding to moderate TBI severity, could be enrolled. This imbalance could be explained by the small proportion of eligible patients in the overall burden of TBI victims, while mild TBI constitutes more than 95%. Patients with a GCS of 9–12 could not be enrolled due to planned surgical intervention and frequent associated polytrauma (both are non-inclusion criteria). Additionally, enrolling unconscious patients often required proxy consent, posing further challenges. In practice, once the recruitment target for patients with a GCS of 13–14 was reached, no patients with a GCS of 9–12 were enrolled. The PTA duration in patients with mild TBI usually does not exceed 1 h; therefore, this measure could not serve as an efficacy criterion in this subgroup. Hence, it was decided to supplement the primary endpoint with one of the secondary endpoints, the proportion of patients who achieved complete recovery (category 8) according to the Glasgow Outcome Scale–Extended (GOS-E) (16) by day 90 (protocol version 6.0 dated August 24, 2023).

The main secondary endpoints were the GOS-E score at 3 months, GOAT score up to day 14 (or at discharge, whichever comes first), Barthel Index (BI) (17) on day 14 (or at discharge, whichever comes first), and mortality on days 14 and 90.

A complete list of endpoints, including tertiary (search) endpoints, is provided in the online Supplementary material.

Safety was assessed by the incidence of adverse events, including the results of physical examination, blood and urine tests, monitoring of vital signs, and results of repeated CT scans (if performed).

### 2.4 Procedures

INRSA/placebo diluted in 200 ml of normal saline was administered intravenously twice daily at 12-h intervals (±2 h) for 10 days.

GOAT was performed by an independent assessor daily on days 2–14. The assessment could be completed early if the GOAT score was >75 for three consecutive days.

The GOS-E was assessed on day 90 ± 7 via telephone interviews. If the patient was not available, the interview could be conducted using the patient's proxy. Structured interviews followed a standardized questionnaire (18).

GCS scores were assessed daily (days 1–14). For a correct assessment, the following conditions must have been met: SpO2 ≥90%, systolic blood pressure ≥90 mmHg, and no exposure to sedatives or other drugs that alter neurological status.

The Barthel Scale score was assessed on day 14 or on the day of discharge. The assessment was based on available clinical information and the patients' answers to the questions.

Computed tomography (CT) was performed on admission (performed as part of routine clinical practice). CT results were classified using the Marshall classification (19) and examined for the presence of focal contusion. Based on the CT results, the baseline predictors of risk of an unfavorable outcome included into the CRASH (The Corticoid Randomization after Significant Head Injury) model (20) were recorded.

### 2.5 Statistical analysis

The sample size calculation was based on a study of progesterone in TBI (21), where complete recovery after 3 months according to the Glasgow Outcome Scale (GOS) was achieved in 47% of patients in the progesterone therapy group and 31% in the placebo group. The GOS-E (the outcome measure in the present study) was developed as a detailed version of the original GOS, where the maximum recovery category (category 5, good recovery) was divided into two categories (categories 7 and 8, low good recovery and upper good recovery) (16). Thus, GOS category 5 includes GOS-E category 8, while both assessments reflect a similar clinical outcome—the maximum possible result achieved, i.e., complete recovery. Thus, the sample size calculation aimed at achieving the highest possible category by GOS-E reflects a conservative approach compared with the GOS assessment used in the study of progesterone. A sample size of 332 patients would achieve 75% power for a 16% difference in proportions of complete recovery and 5% dropout rate. The *Z*-test was used to compare the proportion of complete recovery between the INRSA and placebo groups. The null hypothesis (H0) assumed no difference (*pT*–*pR* = 0), while the alternative hypothesis (H_*a*_) suggested a difference (*pT*–*pR* ≠ 0). The statistical power of 75% was selected to balance scientific rigor with practical feasibility. In this trial, the chosen power reflected realistic limitations in recruitment rate and resources, with transparent acknowledgment that the main risk of lower power concerns interpretability of results rather than patient safety.

Based on the results of the unblinded interim analysis of the data from the first 166 randomized patients, the number of randomized patients could be increased to 430. To control the overall significance level at 2.5% (due to two primary endpoints), the Bonferroni correction was applied, assigning a two-sided α = 0.0125 to each endpoint. For each co-primary endpoint, the group sequential design included two analyses: an interim analysis and a final analysis. Within each endpoint, the allocated α = 0.0125 was further divided equally (50%/50%) between the two analyses, resulting in a two-sided nominal α = 0.00625 for each look. This approach ensured that the overall two-sided α level of 0.0125 per endpoint was preserved. Correspondingly, 98.75% two-sided confidence intervals were used for estimation (100%−1.25%).

At the interim analysis, statistical analysis was performed by an independent DMC. The decision to stop the study due to futility or early demonstration of efficacy or to extend the study was made by an independent DMC based on pre-specified boundaries for *p*-values (efficacy and futility boundaries-−0.0125 and 0.27036—two-sided, respectively), with the alpha level allocated as 50% to the interim and final analyses. Efficacy was considered established if the null hypothesis was rejected by any of the two components of the primary efficacy endpoint. The statistical significance of between-group differences for each criterion was analyzed using a generalized linear model with a binomial distribution and logit link function, taking into account the stratification factor for the difference in the proportions of responders and, if applicable, the impact of the clinical center. The differences between the proportions in the study groups are presented with corresponding 98.75% two-sided confidence intervals. Superiority was considered to be demonstrated if, for at least one of the two primary efficacy criteria, the left border of the calculated confidence interval for the difference in proportions exceeded zero.

Adjusted and unadjusted odds ratios obtained by logistic regression are presented as an additional analysis. Age, baseline GCS score, CT parameters (presence and type of focal contusion, compression of the third ventricle or basal cisterns, midline shift), presence of clinically apparent neurological deficits, clinically significant abnormalities of vital signs, and hemoglobin level were considered as potential factors in the model. Another additional analysis was ordered logistic regression, where GOS-E category at 3 months was a dependent variable, and age, baseline GCS, and CT variables (presence and type of contusion foci, compression of the third ventricle or basal cisterns, midline shift) were the independent variables.

Another additional assessment based on the GOS-E score is the stratification dichotomy method. Patients were categorized into low- and moderate-risk prognostic groups based on the probability of an unfavorable outcome using the CRASH calculator (19) (http://www.crash.lshtm.ac.uk/Risk%20calculator/). For patients with moderate (≥25%) risk of death or severe disability at 6 months a favorable outcome was defined as GOS-E categories 6–8, for patients with low risk (< 25%)—GOS-E category 8.

Between-group comparison for the Barthel Index was performed using the nonparametric Wilcoxon test with the construction of nonparametric confidence intervals.

The primary efficacy endpoint was assessed in the full analysis set (FAS) population, and additional analysis of the primary endpoint in the per-protocol (PP) population is described in the online Supplementary material.

Data analysis was performed using specialized software “The R Project for Statistical Computing” (https://www.r-project.org) version not lower than 4.0.4.

## 3 Results

### 3.1 Disposition and recruitment

**Figure 1:**
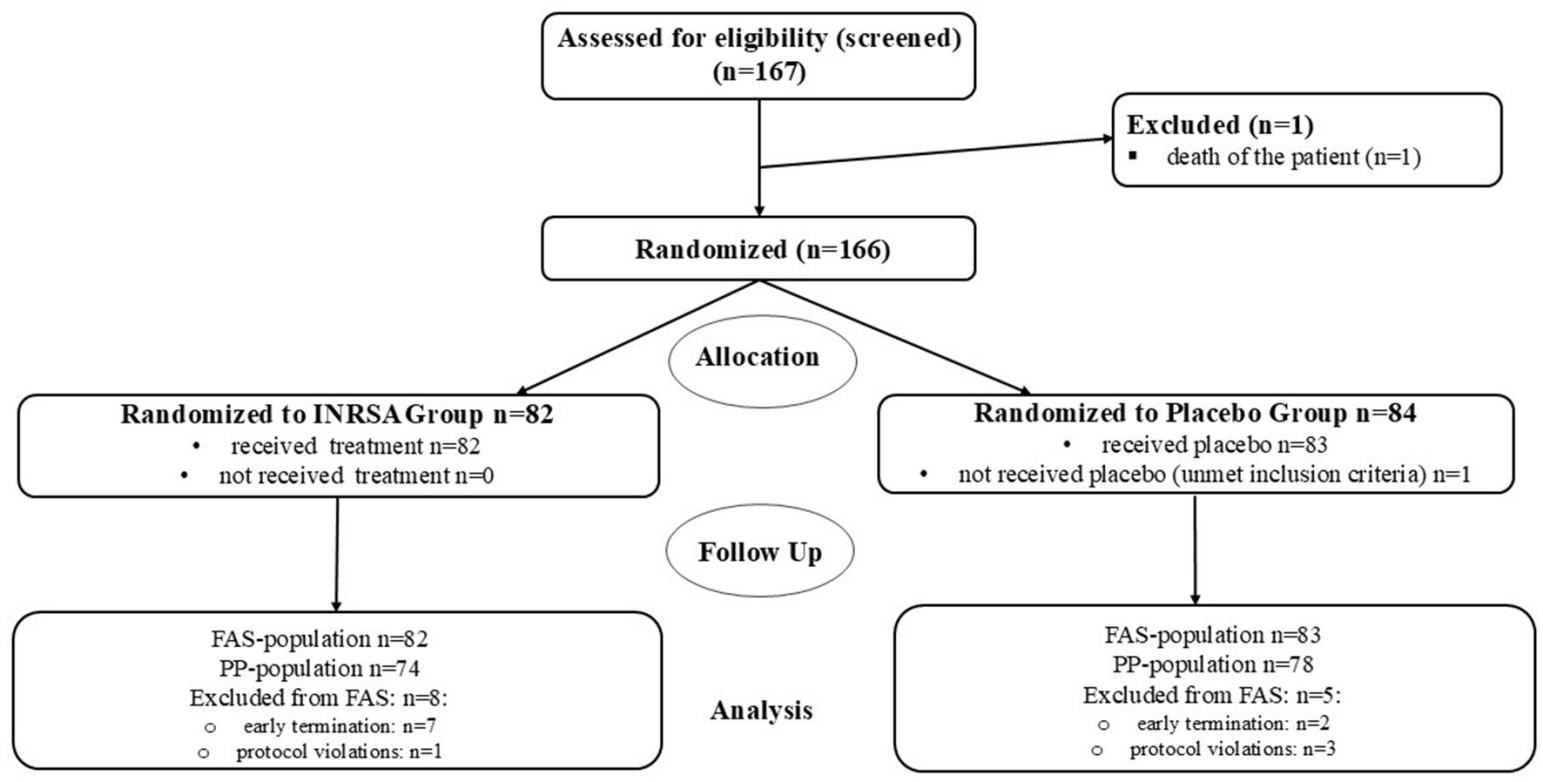
Distribution of patients in the study. INRSA, inosine, nicotinamide, riboflavin, and succinic acid; FAS, full analysis set; PP, per protocol population.

Between November 22, 2020, and May 07, 2024, 166 patients were enrolled (Figure 1), of which 165 were included into the safety population and FAS and 152 into PP population.

All the study patients were Caucasian. Baseline characteristics were balanced between study groups (Table 1). Patients with closed-type TBI predominated in both groups. Twenty-two patients underwent surgery for TBI (debridement and wound suturing) under local anesthesia.

**Table 1:** Baseline characteristics of the study groups (FAS population).

**Characteristic**	**INRSA group (*n* = 82)**	**Placebo group (*n* = 83)**	*P*-value^*^
**Demographic and clinical data**			
Age (years)	42.98 (±11.09)	40.87 (±11.76)	0.245
Male sex	53 (65%)	60 (72%)	0.290
Closed TBI	62 (76%)	61 (73%)	0.755
Open non-penetrating TBI	20 (24%)	22 (27%)	
Surgical procedures for TBI	10 (12%)	12 (14%)	0.819
Concomitant injury outside TBI	4 (5%)	9 (11%)	0.155
Surgery for concomitant injury	0 (0.00%)	2 (2%)	0.497
**Results of CT scanning**			
Diffuse injury I (no visible pathology) by Marshall	26 (32%)	32 (39%)	0.416
Diffuse injury II by Marshall	55 (67%)	51 (61%)	
Non-evacuated mass lesion	1 (1%)	0 (0.00%)	
Parenchymal contusion foci	45 (55%)	37 (45%)	0.186
Skull fractures	43 (52%)	36 (43%)	0.244
Perifocal edema (in the area of the contusion focus)	9 (11%)	4 (5%)	0.161
Midline shift	2 (2%)	1 (1%)	0.620
Local SAH	52 (63%)	61 (73%)	0.368
Diffuse SAH	3 (4%)	2 (2%)	
Pneumocephalus	11 (13%)	5 (6%)	0.109
**Risks of unfavorable outcomes in patients calculated by the**			
**CRASH model**			
Risk of death at 14 days, %	3.81 (±1.97)	3.63 (±1.50)	0.731
Risk of death and disability at 6 months, %	10.34 (±6.30)	9.59 (±5.38)	0.565

FAS, full analysis set; INRSA, inosine, nicotinamide, riboflavin, and succinic acid; TBI, traumatic brain injury; CT, computed tomography; SAH, subarachnoid hemorrhage; CRASH model, corticosteroid randomization after significant head injury model; data presented by mean (±standard deviation) or n (%). ^*^Fisher's exact/Pearson's Chi-squared/Wilcoxon rank sum test.

In 13 patients, concomitant maxillofacial injuries and rib fractures were present, which required surgery in two patients.

All patients had GCS score of 13–14 on admission. Disorders of consciousness were recorded in 76 (93%) patients in the INRSA group and 79 (95%) patients in the placebo group, including confusion, disorientation, agitation, and psychomotor agitation (but not delirium). Neurological symptoms in all included patients corresponded to the clinical diagnosis of mild to moderate cerebral contusion: gaze paresis (three in the placebo group), facial asymmetry (two in the INRSA group and three in the placebo group), limb paresis (one right-sided mild hemiparesis, placebo group), and meningeal signs (26 in INRSA and 34 in the placebo group). Results of the CT scans supported the diagnosis of mild to moderate TBI, where there were no signs of mass effect or raised intracranial pressure (Table 1).

### 3.2 Primary efficacy endpoint

In the interim analysis, PTA regression was observed in 83 (100%) patients in the placebo group and in 78 (99%) patients in the INRSA group, demonstrating the absence of a statistically significant difference (98.75% CI: −0.04; 0.02, *p* = 0.997).

On day 90 ± 7, all patients were interviewed directly, without a need for a proxy. Complete recovery (GOS-E category 8) was achieved in 24 (30%) patients in the placebo group and 55 (73%) in the INRSA group (Table 2). Experimental treatment increased the odds of complete recovery by 6.53 (95% CI: 3.30−13.41) compared to placebo. The difference in the proportion of responders was 44% (95% CI: 0.26; 0.62); thus, since the left border of the confidence interval did not cross zero, it was concluded that the superiority hypothesis was proven based on the GOS-E assessment.

**Table 2:** Proportions and estimated odds ratios for complete recovery (category 8) measures by extended Glasgow outcome scale (GOS-E) at day 90 (FAS population).

**Estimate**	**INRSA (*n* = 75)**	**Placebo (*n* = 81)**	**Difference between proportions (effect size)**	***P*-value**
Proportion of respondents (achieved GOS-E category 8)	0.73 (98.75% CI: 0.59; 0.84)	0.30 (98.75% CI: 0.19; 0.44)	0.44 (98.75% CI 0.26; 0.62)	< 0.0001 (Fisher's exact test)
Unadjusted OR	6.53 (95% CI: 3.30; 13.41)	Reference group	–	< 0.0001
Adjusted OR	6.53 (95% CI: 3.30; 13.41)	Reference group	–	< 0.0001

FAS, full analysis set; INRSA, inosine, nicotinamide, riboflavin, and succinic acid; GOS-E, Glasgow outcome scale-extended; OR, odds ratio; CI were estimated using the Wald method, based on the asymptotic normality of logistic regression parameter estimates.

None of the potential predictors of outcome (clinical site, risk of death at 14 days by CRASH, risk of death and disability at 6 months by CRASH, presence and type of parenchymal contusions, clinically apparent neurological deficit, and hemoglobin level) were statistically significant in the multivariable analysis.

A primary endpoint analysis in the PP population and in the FAS population with the replacement of missing values for the absence of effect is provided in the online Supplementary material. The results obtained were compatible with those of the primary analysis of the FAS population.

Based on the obtained results, the DMC concluded that the efficacy of INRSA had been established, since the null hypothesis was rejected at a significance level of 1.25% for the second primary efficacy criterion (assessment using the GOS-E outcome scale), taking into account the control of type I error. Early termination of the study was recommended because of the early demonstration of its efficacy (Independent Data Monitoring Committee (DMC) meeting report dated 01 July 2024). The futility condition was achieved for the first efficacy criterion based on the GOAT consecutive assessment (*p* = 0.997).

### 3.3 Secondary efficacy endpoints

#### 3.3.1 Barthel index

**Figure 2:**
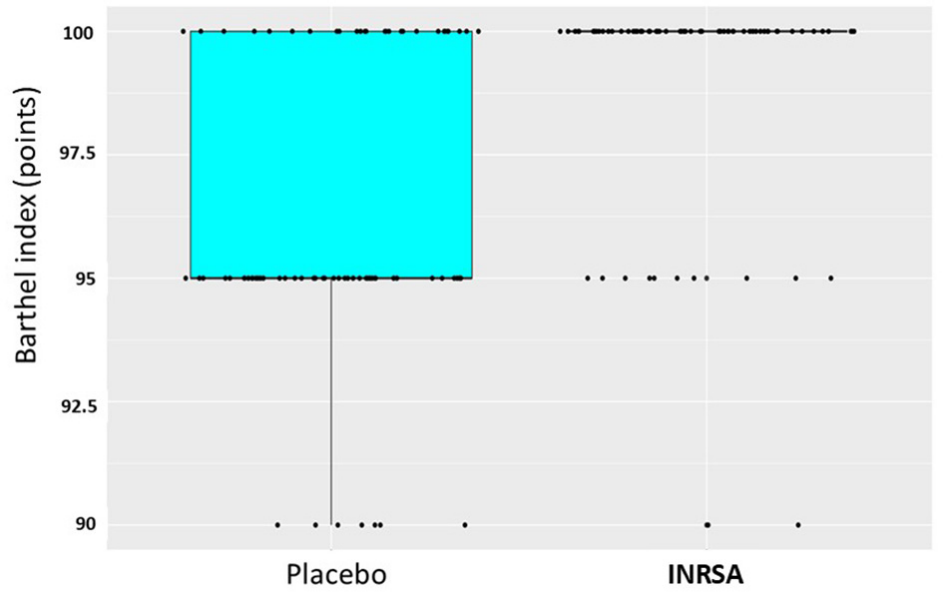
Distribution of Barthel index on day 14/discharge (whatever earlier) in the study groups. INRSA, inosine, nicotinamide, riboflavin, and succinic acid.

The mean Barthel index on day 14 was significantly higher in the INRSA group than in the placebo group (*p* < 0.0001). The shift in the Barthel index on day 14 in the placebo group relative to that in the experimental group was −5.00 with a nonparametric 95% CI (−5.00; −0.00; Table 3; Figure 2).

**Table 3:** Barthel index on day 14/discharge (whatever earlier) in the study groups: shift of distributions by factor.

**Group**	**Mean Barthel index (±SD)**	***W*-value**	***P*-value**	**Shift**	**Nonparametric 95% CI**
Placebo (*n* = 81)	96.23 ± 3.01	1,524.0000	< 0.0001	−5.00	(−5.00; −0.00)
INRSA (*n* = 76)	99.08 ± 2.27				

INRSA, inosine, nicotinamide, riboflavin, and succinic acid; SD, standard deviation; CI, confidence interval.

#### 3.3.2 The result of the assessment of GOS -E category on day 90

In the ordinal regression model, where treatment group, age, initial GCS score, presence and type of contusion foci, presence of compression of the third ventricle or basal cisterns, midline shift, clinically significant neurological deficit, abnormal vital signs, hemoglobin level were considered as potential factors, only the treatment group was a significant factor. The odds of a higher level of recovery were 6.82 times higher in the INRSA group vs. placebo group (Table 4). Full distribution of the GOS-E estimates at Day 90 is given in the Supplementary material.

**Table 4:** Results of ordinal logistic regression for the higher GOS-E category on day 90.

**Factor**	**Estimate**	**OR**	**95% CI**	***P*-value**
Group (INRSA/placebo)	1.92	6.82	(3.44; 13.51)	< 0.0001
**Intercept**				
GOS-E level 6 (upper moderate disability) | GOS-E level 7 (low good recovery)	−1.37			< 0.0001
GOS-E level 7 (low good recovery) | GOS-E level 8 (upper good recovery)	0.90			0.0002

GOS-E, Glasgow outcome scale-extended; INRSA, inosine, nicotinamide, riboflavin, and succinic acid; OR, odds ratio.

#### 3.3.3 . Proportions of favorable outcome (sliding dichotomy analysis)

Based on the division into prognostic groups depending on the risk according to the CRASH model, the odds of a favorable outcome were 6.08 times higher in the INRSA group than in the placebo group (Table 5).

**Table 5:** Comparison of the proportion of patients with favorable outcome (based on the division into prognostic groups depending on the risk according to the CRASH model) in the study groups.

**Estimate**	**Placebo (*N* = 81)**	**INRSA (*N* = 75)**
Mean CRASH-calculated risk of unfavorable outcome at 6 months (±SD)	9.59 (±5.38)	10.34 (±6.30)
Favorable outcome	25 (31%)	55 (73%)
Unfavorable outcome	56 (69%)	20 (27%)
Odds ratio	6.0787	
Fisher's exact test, *p*-value	< 0.0001	

INRSA, inosine, nicotinamide, riboflavin, and succinic acid; CRASH, the corticoid randomization after significant head injury.

No statistical significance was observed for the other secondary endpoints.

### 3.4 Analysis of safety

The safety analysis included a total of 165 patients. Thirty-six adverse events (AE) were identified in 23 patients (14%): 23 AEs in 14 patients (17%) in the INRSA group, 13 AEs in nine patients (11%) in the placebo group.

In three (4%) patients in the INRSA group, five AEs had at least a possible relation to experimental treatment: itching (*n* = 1), headache (*n* = 2), and erythema at the site of administration (*n* = 2). AEs associated with the use of the experimental medication were expected (described in the medicinal product label), transient, and of mild severity.

No deaths occurred during the study period. After treatment completion, two serious adverse events (SAE) were recorded in two (1%) patients. One patient (1 %) in the INRSA group had a head wound during the follow-up period. One (1%) patient in the placebo group was hospitalized for elective surgery (internal fixation of facial bone fracture). These SAEs were not related to the experimental treatment.

## 4 Discussion

In a multicenter randomized controlled trial, experimental treatment with INRSA was associated with an improvement in the mild TBI outcome. In previous studies, several pharmaceutical interventions have demonstrated potential efficacy (22), but clinical trials have yielded inconclusive results; consequently, no pharmaceutical neuroprotection is currently recommended (1, 8, 23). To our knowledge, this is the first late-phase clinical trial with positive results. Effect size, as measured by the proportion of patients who achieved complete recovery (category 8 by GOS-E) on day 90 after TBI, was large enough (at least 26% higher in the treatment group than in the placebo group) to demonstrate early efficacy in the interim analysis. Although the difference between upper good recovery and GOS-E categories 6–7 by may seem subtle, we assume that this difference is considerable for an individual patient since complete recovery implies the absence of any significant consequences of TBI and return to professional and social activities. The proportion achieved in the experimental group (73%) was higher than the previously reported 56%−57% of complete recovery (GOSE = 8) after TBI at 6 months (24, 25), although direct comparison is hardly possible because of differences in population and clinical practices. On the contrary, in the control group the rate of disability (70%) was higher than previously reported 54% in the recent meta-analysis of outcomes in mild TBI (26); this finding may be explained by early assessment on day 90 in the present study compared to 3–6 months follow-up in most trials included into the meta-analysis.

We failed to demonstrate improvement of PTA, as assessed by GOAT, in the acute period of TBI. GOAT score provides an objective assessment of early cognitive recovery and correlates with the long-term functional outcome, return to work, psychosocial functioning, and distress (27–29). Regression of PTA in patients with mild TBI usually occurs early, which allows to perform efficacy assessments before the patient is discharged from hospital. Mainly for this reason, the GOAT scale was initially planned as the single primary endpoint, in view of high risk of loss to follow-up in patients with social deviations which correlate with TBI (30). In reality, recruitment happened to be limited to mild TBI (GSC 13–14) because of low proportion of patients with GCS 9–12 and occurrence of exclusion criteria (extracranial injuries, CT findings, or major surgery) in these patients. The duration of PTA in patients with mild TBI mostly does not exceed 1 h, therefore the duration of PTA could not demonstrate efficacy in this subgroup. While ceiling effect was achieved in immediate GOAT assessment, the clinical benefit according to GOS-E was observed at a later time after the TBI. GOS-E is a recognized tool for assessing efficacy in clinical trials in patients with TBI and is widely used in international clinical trials (15). This result may be interpreted as more relevant, compared to initial aim at GOAT score improvement, since GOS-E is a direct measurement of disability, while GOAT is an indirect correlate of the long-term outcome.

Short-term recovery of functional activity was measured by Barthel index on day 14, and the result corresponds to the result of 3-months GOS-E assessment. Between-group difference by BI was subtle—shift by 5%—yet statistically significant. This finding is appropriate for patients with mild to moderate TBI, in whom severe disability, corresponding to low BI scores, is expected to be rare (25).

The study population did not include patients with moderately severe and severe TBI. Many attempts to improve the outcome of severe TBI yielded no clinical effect (8, 31); the reasons could be severity of secondary injury, surgery-related factors, and extracranial injuries. Our population was chosen to maximize the chance to demonstrate the effect of pharmacological intervention, i.e., patients who need hospital admission but do not need surgery/ICU. In fact, instead of the initially planned population of patients with moderate TBI we studied mild TBI subjects; this phenomenon may reflect the huge quantitative predominance of mild severity in this condition. Outcomes in this subpopulation presuppose low mortality, but deferred disability in terms of impairment of cognitive functions and difficulties in daily activities (3–5). For this reason, we assume that the result of the study is clinically relevant, despite the unprompted change of target condition from moderate to mild TBI.

The limitations of the present study are associated with its single blind design, low representation of moderate to severe TBI which complicates generalizability of results, a small number of participants who were the residents of the two participating countries. However, the study groups were well-balanced, and the result was consistent for the primary and secondary outcomes. GOS-E was assessed via telephone interview, which may be a potential source of uncertainty.

## 5 Conclusion

In the setting of a multicenter randomized controlled clinical trial, experimental treatment with INRSA was associated with improvement of mild TBI outcome, which represents a new potential option for pharmacological treatment of mild head injury. Further studies, larger in size and involving TBI of various severity, might be of research interest and clinical demand.

## Data Availability

The original contributions presented in the study are included in the article/Supplementary material, further inquiries can be directed to the corresponding author.
