# Supplementary material for: A multicenter, international, randomized, single-blind, placebo-controlled study of the efficacy and safety of inosine-nicotinamide-riboflavin-succinic acid in the acute period of traumatic brain injury in adults
Source: Front Neurol. 2026 Jan 2;16:1683976. doi: 10.3389/fneur.2025.1683976 (PMC12807964; doi:10.3389/fneur.2025.1683976)
Supplement: Supplementary file 1 [file Table_1.docx]

Supplementary Material

# Supplementary Data

**Eligibility criteria**

Inclusion Criteria:

1. Age from 18-60 (inclusive).

2. Clinical diagnosis of TBI, cerebral contusion of moderate severity without compression.

3. The written consent of the legal representative or the decision of the council to include the patient in the study.

4. Possibility of a full assessment of eye opening, speech and motor response by GCS.

5. GCS at the time of inclusion 9 - 14 (inclusive).

6. Time of initiation of study drug therapy within 24 hours after the estimated or determined time of injury.

7. The presence of post-traumatic amnesia, confusion and disorientation.

8. Absence of indications for neurosurgery or other surgical intervention under general anesthesia.

9. Normal brain CT scan, or the presence of subarachnoid hemorrhage and / or contusion foci of I-III types according to Kornienko and / or limited or diffuse cerebral edema.

10. The expected duration of hospital stay >= 10 days.

11. Absence of a disabling neurological or mental illness, information about the patient's disability prior to injury.

12. Possibility to perform all procedures stipulated by the study protocol

Non-inclusion Criteria:

1. The need to use the therapy prohibited by the study protocol.

2. Concomitant injury, except for cases of damage to the skeleton, soft tissues, internal organs, which do not require (1) surgical intervention under general anesthesia, and (2) are not an independent indication for hospital treatment.

3. Past / planned surgical intervention for the current episode of trauma under general anesthesia.

4. Penetrating open TBI.

5. Presence of the following lesions on the results of computed tomography (CT) of the brain performed prior to the patient's randomization:

a. epidural hematoma or subdural hematoma;

b. evidence of a previous head injury based on CT results;

c. type IV contusion foci according to Kornienko's classification.

6. Presence of any of the following risk factors for secondary brain injury at any time after TBI: hypoxia (SpO2 <90% based on pulse oximetry results); hypotension (systolic blood pressure <90 mm Hg) or shock;hypothermia (body temperature <35 ° C); clinical signs of respiratory failure, the need for mechanical ventilation.

7. Drug addiction.

8. Alcohol in saliva >=2 ‰ or a previous diagnosis of alcohol dependence.

9. Depression of consciousness, presumably resulting from other reasons (for example, alcohol, drugs, drugs, poisonous substances).

10. The presence of aphasia due to focal brain damage, which prevents communication with the researcher.

11. Status epilepticus at the time of admission to the hospital or condition after an epileptic seizure.

12. Pregnant and lactating women.

13. Availability of information about concomitant chronic disease in the stage of decompensation.

14. Intolerance to the components of CYTOFLAVIN®, anamnestic data on drug allergy to succinic acid, riboflavin, inosine, or nicotinamide.

15. Severe renal or heart failure requiring restriction of the volume of injected fluid.

16. The presence of a condition or disease that, in the opinion of the investigator, jeopardizes the patient's safety if the patient participates in the study, or may interfere with the performance of examination procedures, an objective assessment of the patient's condition, or distort the assessment of the outcome of TBI.

17. Participation in any clinical study less than 3 months before the start of the study.

18. Patients who are employees of the research center and their families.

19. Language barrier.

20. Availability of information that the patient is a stateless person or a citizen of another state

Exclusion Criteria:

1. Withdrawal of informed consent or refusal to continue participating in the study.

2. The need to perform neurosurgery or other surgical intervention under general anesthesia.

3. Alcohol delirium.

4. Failure of the patient to comply with the protocol procedures.

5. The need for a course (for 3 or more consecutive days) of prohibited treatment.

6. Any condition of the patient in which, in the opinion of the investigator, the patient's continued participation in the study jeopardizes the patient's safety or requires the patient's withdrawal from the study.

**Study endpoints**

Primary endpoint

• The proportion of patients with regression of post-traumatic amnesia by day 7 of treatment, defined as having a Galveston scale score of more than 75 points for 3 consecutive days up to and including day 7 of treatment,

or

• the proportion of patients who achieved complete recovery (category 8) according to the extended Glasgow Outcome Scale (GOS) by Day 90 from the start of the study

Secondary endpoints

• Expanded Glasgow Outcome Scale (GOS) score at month 3

• Galveston score up to day 14 of the study (or discharge, whichever comes first)

• Barthel Index at day 14 of the study (or discharge, whichever comes first)

• Mortality at days 14 and 90 of the study

Tertiary (search) endpoints

• Time (days) to confirmed resolution of amnesia, defined as the first occurrence of a Galveston score greater than 75 in a series of three such scores on three consecutive days prior to study day 14 (or discharge, whichever comes first)

• Glasgow Coma Scale (GCS) score prior during 14 days (or discharge, whichever comes first)

• Time to patient regaining consciousness, defined as the first occurrence of a GCS score of 15

• Change in head computed tomography (CT) scan results on repeat CT scans compared to admission CT scan (if patients undergo repeat head CT scans)

• Change in serum S100β protein level on day 10 of treatment compared to baseline

# Supplementary Figures and Tables

**Additional analysis of primary endpoint**

The primary efficacy endpoint was assessed in the FAS population, with a supportive analysis of the primary efficacy endpoint also conducted in the PP population. An additional analysis was conducted in the FAS population with missing primary endpoint values replaced by 0 – no response (e.g., in case of early termination or dropout the subject was considered a non-responder).

Supplemental table 1. Between-group comparison of proportions of patients with regression of posttraumatic amnesia by day 7 of treatment (interim analysis; PP population)

| **Estimate** | **Placebo (n = 78)** | **INRSA (n = 74)** | **p** |
| --- | --- | --- | --- |
| **Number of responders, n(%)** of patients with regression of posttraumatic amnesia by day 7 of treatment | 78 (100%) | 74 (100%) | >0.9999 |
| Unadjusted OR (95% CI) | Reference group | 1.00 (-; -) | >0.9999 |

Supplemental table 2. Proportions and estimated odds ratios for complete recovery (category 8) measures by extended Glasgow Outcome Scale (GOS-E) at Day 90 (interim analysis; PP population)

|  | **Placebo**  **(n=78)** | **INRSA**  **(n=74)** | **Difference between proportions** | **p** |
| --- | --- | --- | --- | --- |
| Proportion of respondents (achieved GOS-E category 8) (98.75%CI) | 0.28  (0.17; 0.42) | 0.73  (0.58; 0.84) | 0.45  (0.27; 0.63) | <0.0001 |
| Unadjusted OR (95%CI) | Reference group | 6.87 (3.43; 14.30) |  | <0.0001 |

Supplemental table 3. Between-group comparison of proportions of patients with regression of posttraumatic amnesia by day 7 of treatment with replacement of missing values ​​for the absence of effect (failure to achieve regression of post-traumatic amnesia (interim analysis; FAS population)

| **Estimate** | **Placebo (n = 83)** | **INRSA (n = 82)** | **p** |
| --- | --- | --- | --- |
| **Number of responders, n(%)** of patients with regression of posttraumatic amnesia by day 7 of treatment | 83 (100.00%) | 78 (95.12%) | >0.0587 |
| Unadjusted OR (95% CI) | Reference group | 0.00 (-; -) | 0.9954 |

Supplemental table 4. Proportions and estimated odds ratios for complete recovery (category 8) measures by extended Glasgow Outcome Scale (GOS-E) at Day 90, with replacement of missing values ​​for the absence of an effect (failure to achieve complete recovery). (interim analysis; FAS population)

|  | **Placebo**  **(n=83)** | **INRSA**  **(n=82)** | **Difference between proportions** | **p** |
| --- | --- | --- | --- | --- |
| Proportion of respondents (achieved GOS-E category 8) (98.75%CI) | 0.29  (0.18; 0.43) | 0.67  (0.53; 0.79) | 0.38  (0.20; 0.56) | <0.0001 |
| Unadjusted OR (95%CI) | Reference group | 5.01 (2.62; 9.85) |  | <0.0001 |

Supplemental table 5. Distribution of the extended Glasgow Outcome Scale (GOS-E) at Day 90.

|  | **INRSA (N=75)** | **Placebo (N=81)** | **Total (N=156)** |
| --- | --- | --- | --- |
| Category 6: Upper Moderate Disability | 2 (3%) | 17 (21%) | 19 (12%) |
| Category 7: Lower Good Recovery | 18 (24%) | 40 (49%) | 58 (37%) |
| Category 8: Upper Good Recovery | 55 (73%) | 24 (30%) | 79 (51%) |

## Supplementary Figures


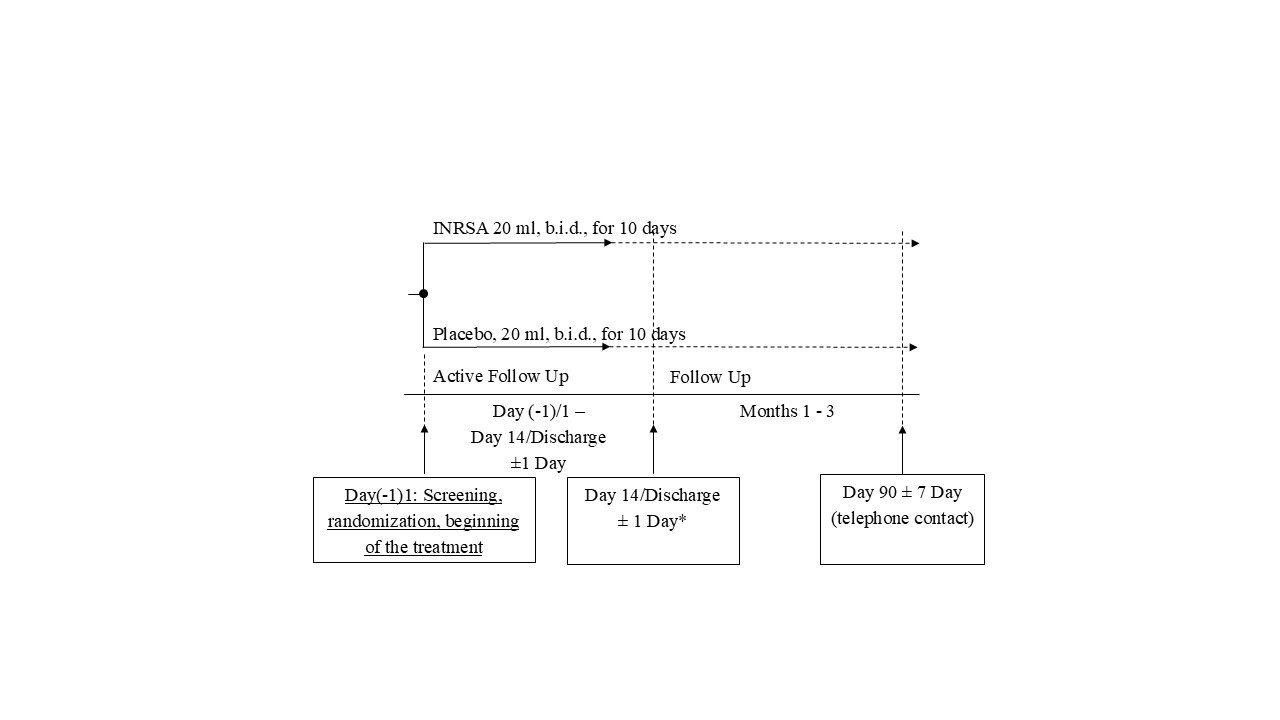


**Supplemental figure 1. Study design**
